# Reliability and construct validity of the Italian EQ-5D-5 L in Parkinson’s disease: a cross-sectional study

**DOI:** 10.1186/s41687-026-01174-2

**Published:** 2026-08-10

**Authors:** Giovanni Sellitto, Andrea Broglio, Emanuele Amadio, Francescaroberta Panuccio, Andrea Marini Padovani, Anna Berardi, Giovanni Galeoto, Ilaria Ruotolo

**Affiliations:** 1 Department of Human Neurosciences, Sapienza University of Rome, Rome, Italy; 2 Department of Public Health and Infectious Disease, Sapienza University of Rome, Rome, Italy; 3 Sapienza University of Rome, Rome, Italy; 4 Istituto Neurologico Mediterraneo, Pozzilli, Italy

**Keywords:** EQ-5D-5L, Parkinson’s disease, Health-related quality of life, Patient-reported outcome measures, Short-term reproducibility, Construct validity

## Abstract

**Background:**

Parkinson’s disease (PD) substantially affects health-related quality of life (HRQoL). The EQ-5D-5 L is a preference-based patient-reported outcome measure that supports cross-condition comparison and health-economic evaluation, but its measurement properties have not been established in people with PD in Italy. This study evaluated short-term reproducibility and construct validity through a priori hypothesis testing; score distributions and inter-dimensional associations were also described.

**Methods:**

This cross-sectional psychometric study included a 48-hour retest component. Participants completed the EQ-5D-5 L, the Parkinson’s Disease Questionnaire-39 (PDQ-39), and the 12-item Short Form Health Survey (SF-12). Short-term reproducibility was assessed using quadratic-weighted Cohen’s kappa and exact agreement for the five dimensions and single-measures, two-way random-effects, absolute-agreement intraclass correlation coefficients for the EQ-Index and EQ-VAS. Construct validity was examined using prespecified directional and magnitude hypotheses tested with Spearman correlations; Pearson correlations were used as sensitivity analyses. Post hoc analyses examined routine-care clinical measures and ordered motor-severity categories.

**Results:**

Eighty participants completed the questionnaires, and 60 underwent retesting. Fifty-nine distinct health states were observed, with one participant reporting full health. Agreement was high for the EQ-Index (ICC = 0.901, 95% CI 0.840–0.940) and EQ-VAS (ICC = 0.938, 95% CI 0.899–0.963), whereas dimension-level kappa ranged from 0.632 to 0.916. All 20 directional hypotheses were confirmed, but only 6 of the 16 prespecified magnitude criteria were met. The strongest associations were observed with PDQ-39 Mobility (ρ = −0.786 and − 0.781), PDQ-39 Activities of Daily Living (ρ = −0.671 and − 0.685), and the SF-12 Physical Component Summary (ρ = 0.730 and 0.639) for the EQ-Index and EQ-VAS, respectively. In post hoc analyses, both summary scores declined monotonically across externally defined mild, moderate, and severe motor-severity categories (both *p* < 0.001).

**Conclusions:**

The Italian EQ-5D-5 L showed high short-term reproducibility for its summary scores and evidence supporting selected aspects of construct validity, particularly convergence with physical functioning and daily activities. Evidence for discrimination from more distant HRQoL domains was limited, and the post hoc clinical findings require prospective confirmation. The instrument may complement disease-specific measures in group-level research and health-economic evaluation, but these findings do not support its use for monitoring change in individual patients.

**Supplementary Information:**

The online version contains supplementary material available at https://doi.org/10.1186/s41687-026-01174-2.

## Background

Parkinson’s disease (PD) is a chronic, progressive neurodegenerative disorder characterised by a combination of motor features—bradykinesia, rigidity, resting tremor and postural instability—and a wide range of non-motor symptoms, including autonomic dysfunction, sleep disturbance, mood disorders and cognitive decline [1, 2]. It is among the most common neurodegenerative diseases worldwide, and its prevalence is rising with population ageing, placing a growing burden on health-care systems [3]. Beyond their direct clinical consequences, both motor and non-motor symptoms progressively restrict independence and social participation, eroding the health-related quality of life (HRQoL) of people with PD and their caregivers [3, 4]. In Italy, where the population is among the oldest in Europe, PD is a major and increasing public-health concern, and Italian-language PROMs such as the PDQ-39 have been validated to support its assessment [4, 5].

HRQoL has become a central outcome in the management of chronic neurological conditions because it captures the patient’s own perspective on the impact of disease on daily functioning and well-being [6]. Two complementary families of PROMs serve this purpose. Disease-specific instruments are designed to be more sensitive to the problems that matter most in a given condition; in PD, the most widely used is the Parkinson’s Disease Questionnaire-39 (PDQ-39), a non-preference-based measure whose summary index is obtained by averaging eight domain scores [7]. Generic, preference-based quality-of-life (PbQoL) measures, by contrast, describe health on a common metric across conditions and generate utility values suitable for health-economic evaluation [8].

The EQ-5D, developed by the EuroQol Group, is the most widely used generic PbQoL instrument and has been applied across diseases and populations since the 1980s [8, 9]. Its five-level version, the EQ-5D-5 L, was introduced to reduce the ceiling effects and limited discriminative capacity of the original three-level version (EQ-5D-3 L); head-to-head comparisons have confirmed improved measurement properties, reduced ceiling effects and greater sensitivity to small health changes [10–12]. The instrument is now available in more than 130 languages, and interim crosswalk algorithms allow EQ-5D-3 L value sets to be applied where country-specific EQ-5D-5 L weights are not yet available [11, 13]. In PD, the EQ-5D has repeatedly proved feasible and valid: Schrag first demonstrated that it reflects disease severity and complications, and subsequent studies validated the EQ-5D-5 L against the PDQ-8 in a Mexican cohort and against the 15-D instrument in a Spanish PD sample, while a recent meta-analysis summarised EQ-5D utility values across PD populations [14–17].

Despite this evidence, the psychometric performance of the EQ-5D-5 L has not been formally established in Italian people with PD. A validated Italian EQ-5D-5 L value set and recent Italian general-population norm data are now available, enabling utility-based and comparative analyses in this setting [18–20]. Moreover, the EQ-5D-5 L has recently been validated in other Italian neurological populations—most recently in multiple sclerosis—supporting its extension to PD [21].

Characterising selected measurement properties in this population would support more informed use of the EQ-5D-5 L in Italian PD research and health-economic evaluation. The primary aim of the present study was therefore to evaluate its short-term reproducibility and construct validity through a priori hypothesis testing. Score distributions and inter-dimensional associations were also described. Content validity, structural validity, cross-cultural validity and measurement invariance, criterion validity, and responsiveness were not evaluated in the present study.

## Methods

### Study design

This was a cross-sectional psychometric study with a short-term retest component, designed to evaluate selected measurement properties of the Italian EQ-5D-5 L in people with PD. The cross-sectional component was used to examine score distributions and construct validity, while the retest component assessed short-term reproducibility. Responsiveness to change was not evaluated. The study was conducted and is reported in accordance with COSMIN guidance [22].

### Participants and eligibility criteria

Participants were consecutively enrolled at the Department of Human Neurosciences of Sapienza University of Rome between February and October 2024. Eligible participants were adults with a neurologist-confirmed diagnosis of PD, sufficient proficiency in Italian to understand and complete the study questionnaires, and the capacity to provide written informed consent. Participants were excluded if the treating neurologist, based on the clinical record and clinical assessment, identified: (i) documented dementia or cognitive impairment considered likely to prevent independent and reliable questionnaire completion; (ii) a psychiatric disorder of sufficient severity to compromise comprehension, judgement, or the reliability of self-report; (iii) a neurological condition other than PD, such as stroke, with residual cognitive, communicative, or functional consequences relevant to questionnaire completion; or (iv) a sensory or language impairment that precluded valid self-report.

Eligibility was determined clinically at enrolment. No prespecified numerical cognitive-screening threshold was applied, and routine Mini-Mental Parkinson (MMP) scores were not systematically available or reviewed when eligibility was assessed. The questionnaires were completed independently, without proxy responses or item-by-item assistance from caregivers or study personnel. All participants were informed that participation was voluntary and that their data would be anonymised and handled confidentially [23, 24].

A formal a priori power calculation was not performed; sample size was instead guided by established recommendations for psychometric validation studies, which suggest between 2 and 20 participants per item, while acknowledging that reliability coefficients under the null and alternative hypotheses can be difficult to pre-specify [25, 26]. Recruitment was therefore planned to achieve a sample broadly consistent with these recommendations and with comparable single-centre PROM studies.

### Outcome measures

The EQ-5D-5 L descriptive system comprises five dimensions—mobility, self-care, usual activities, pain/discomfort and anxiety/depression—each rated on five severity levels (no, slight, moderate, severe and extreme problems or unable to), generating 3,125 possible health states coded from full health (11111) to the worst state (55555) [27]. Each profile was converted into a single index value using the Italian EQ-5D-5 L value set, derived from composite time trade-off and discrete-choice experiment data, with index values ranging from − 0.571 (55555) to 1 (11111) [18, 19]. The questionnaire also includes a visual analogue scale (EQ-VAS) on which respondents rate their current health from 0 (worst imaginable) to 100 (best imaginable) [28].

The PDQ-39 is the most widely used disease-specific HRQoL measure for PD and assesses eight domains (mobility, activities of daily living, emotional well-being, stigma, social support, cognition, communication and bodily discomfort) through 39 items scored from 0 (never) to 4 (always); domain and summary index scores range from 0 (best) to 100 (worst), with higher scores indicating poorer HRQoL [29]. The Italian version has been validated. The SF-12 is a short generic health survey derived from the SF-36 that yields a Physical Component Summary (PCS-12) and a Mental Component Summary (MCS-12) and has been used as a HRQoL comparator in PD [30].

As part of routine clinical care, participants attending the Department underwent assessment with the Movement Disorder Society–Sponsored Revision of the Unified Parkinson’s Disease Rating Scale (MDS-UPDRS), comprising Part I (non-motor experiences of daily living), Part II (motor experiences of daily living), Part III (motor examination), and Part IV (motor complications), as well as with the Mini-Mental Parkinson (MMP), a 32-point cognitive screening instrument specifically developed for people with PD [31, 32]. These assessments were conducted by an experienced movement-disorders neurologist, during the same visit as questionnaire administration, and MDS-UPDRS Part III was administered in the ON medication state. Because the MDS-UPDRS and MMP were collected for clinical purposes rather than according to a prespecified research assessment schedule, their availability varied across participants. Analyses involving these measures were therefore conducted on an available-case basis, including only participants with recorded data for the relevant assessment.

### Statistical analysis

All analyses were performed with IBM SPSS Statistics (version 29). Descriptive statistics summarised the demographic and clinical characteristics of the sample; continuous variables were expressed as means and standard deviations and categorical variables as frequencies and percentages.

The distributions of the EQ-Index and EQ-VAS were examined using skewness, kurtosis, and the Shapiro–Wilk test. Interpretability was described by reporting the number of distinct EQ-5D-5 L health states observed, the proportions of participants reporting full health (11111) and the worst possible health state (55555), and the frequency of negative EQ-Index values. Rank-based methods were used where the distributional assumptions for parametric analyses were not adequately met [33].

Cronbach’s alpha, corrected dimension–total correlations, and alpha following deletion of each dimension were calculated descriptively to characterise associations among the five EQ-5D-5 L dimensions. These coefficients were not interpreted as evidence of internal-consistency reliability or unidimensionality, because such an interpretation assumes a unidimensional reflective measurement model, whereas the EQ-5D-5 L is a preference-weighted health-state classification system whose dimensions may have both formative and reflective relationships [34, 35]. Structural validity was not evaluated. A conventional single-factor exploratory or confirmatory model was not considered an adequate representation of the descriptive system, while alternative mixed formative–reflective modelling was outside the prespecified design and sample size of the study.

Short-term reproducibility was evaluated in a subsample who completed the EQ-5D-5 L a second time after a 48-hour interval. Because the five dimensions of the descriptive system are ordinal, agreement at dimension level was quantified with quadratic-weighted Cohen’s kappa and with the proportion of exact agreement. For the continuous EQ-Index and EQ-VAS, agreement was quantified with the intraclass correlation coefficient (ICC), estimated using a two-way random-effects model with absolute agreement and reported as single-measures values with 95% confidence intervals, since these are the relevant estimates for a single administration of the instrument; coefficients ≥ 0.70 were regarded as satisfactory [36]. Systematic differences between administrations were examined primarily using the Wilcoxon signed-rank test, with paired-samples t-tests conducted as sensitivity analyses.

The standard error of measurement (SEM) was calculated as SD × √(1 − ICC) and the individual-level smallest detectable change as SDC = 1.96 × √2 × SEM. The 48-hour interval was selected to minimise the likelihood of a genuine change in the underlying health state in a progressive condition; no external anchor of clinical stability, such as a global rating of change, was administered.

Construct validity was examined through a priori hypothesis testing, as recommended by COSMIN [22]. Because higher EQ-5D-5 L and SF-12 scores indicate better health, whereas higher PDQ-39 scores indicate poorer HRQoL, positive associations were expected with the two SF-12 component scores and negative associations with all eight PDQ-39 domains. Each comparator was examined against both the EQ-Index and EQ-VAS, yielding 20 prespecified directional hypotheses. Strong associations (absolute coefficient ≥ 0.50) were hypothesised with the SF-12 Physical Component Summary and the PDQ-39 Mobility and Activities of Daily Living domains; moderate associations (0.30–0.49) with the SF-12 Mental Component Summary and PDQ-39 Emotional Well-being; and weak associations (< 0.30) with the PDQ-39 Stigma, Social Support, and Communication domains [33]. Accordingly, both direction and magnitude were prespecified for 16 associations. For the PDQ-39 Cognition and Bodily Discomfort domains, only the expected negative direction was prespecified, and the observed magnitudes were interpreted descriptively.

Spearman’s rank correlation coefficient was used as the primary analysis because the distributional assumptions for Pearson correlation were not adequately met, particularly for the EQ-Index. Pearson product–moment correlations were computed as a sensitivity analysis. The direction and magnitude of each association were evaluated separately for the EQ-Index and EQ-VAS against the prespecified hypotheses. All tests were two-tailed, with statistical significance set at *p* < 0.05.

Post hoc clinical analyses were undertaken in response to peer review using available-case data from the clinical assessments recorded during routine care. Spearman’s rank correlation coefficients were calculated between the EQ-Index and EQ-VAS and MDS-UPDRS Parts I–IV and the MMP. Correlation tests were two-tailed, and coefficients were interpreted according to the same thresholds used for the primary construct-validity analyses.

To examine whether EQ-5D-5 L scores varied across externally defined levels of motor severity, MDS-UPDRS Part III scores were categorised using the thresholds proposed by Martínez-Martín and colleagues: mild (≤ 32), moderate (33–58), and severe (≥ 59) [37]. For this post hoc analysis, the literature-informed directional expectation was that both the EQ-Index and EQ-VAS would decrease as motor severity increased. Because the categories were ordered, the Jonckheere–Terpstra test for ordered alternatives was used as the principal analysis, while the Kruskal–Wallis test and one-way analysis of variance were conducted as supplementary omnibus analyses. No expected effect magnitude had been prespecified; the observed magnitude was therefore described using eta-squared (η²), the mean difference between the mild and severe groups with its 95% confidence interval, and Hedges’ g with its 95% confidence interval. The analysis was not part of the original analysis plan and was interpreted as exploratory and hypothesis-generating.

Exploratory subgroup analyses examined associations between EQ-5D-5 L scores and selected demographic and clinical characteristics. Because directional hypotheses had not been prespecified for these variables, the analyses were not considered confirmatory evidence of known-groups validity. Two-level grouping variables—gender, employment status, physiotherapy engagement, and body mass index category—were compared using the Mann–Whitney U test, with independent-samples Student’s t-tests conducted as sensitivity analyses. Educational level was examined using the Kruskal–Wallis test, with one-way analysis of variance conducted as a sensitivity analysis. Homogeneity of variance was assessed using Levene’s test, and Welch’s correction was applied to the parametric analyses when the assumption of equal variances was not met. Time since diagnosis was analysed as a continuous variable using Spearman’s rank correlation coefficient, with Pearson’s correlation conducted as a sensitivity analysis. All tests were two-tailed, with statistical significance set at *p* < 0.05. No adjustment for multiple comparisons was applied. Comparisons for which the primary non-parametric analysis and the corresponding parametric sensitivity analysis led to different conclusions were considered inconclusive and were not used to support the construct-validity findings.

## Results

### Participant flow

A prospective screening log was not maintained; consequently, the number of patients assessed for eligibility and the reasons for non-enrolment could not be reconstructed. Eighty people with PD were consecutively enrolled between February and October 2024. All enrolled participants completed the EQ-5D-5 L, the PDQ-39, and the SF-12 and were included in the primary analyses, with no post-enrolment exclusions or missing data for these questionnaires. The EQ-5D-5 L was re-administered after 48 h to the first 60 participants enrolled. The remaining 20 participants were not scheduled for retesting; therefore, their absence from the retest analysis did not represent loss to follow-up. Routine-care MDS-UPDRS data were available for 39 participants for Parts I and II and for 40 participants for Parts III and IV, while MMP data were available for 50 participants. Analyses involving these clinical measures were restricted to participants with available data.

### Sample characteristics

The demographic and clinical characteristics of the cohort are summarised in Table 1. Participants had a mean age of 70.6 ± 8.9 years (range 51–87); 46 (57.5%) were male and 34 (42.5%) female. With respect to education, 3 participants (3.75%) had completed elementary school, 16 (20.0%) middle school, 36 (45.0%) held a high-school diploma, 5 (6.25%) a bachelor’s degree, and 20 (25.0%) a master’s degree. Thirty-nine participants (48.75%) were undergoing physiotherapy, and 64 (80.0%) were not in employment at the time of assessment, a group consisting largely of retired participants given the mean age of the cohort. Thirty-nine participants (48.75%) were of normal weight and 41 (51.25%) were overweight. Time since diagnosis ranged from 1 to 26 years (mean 7.1 ± 5.2): 51 participants (63.75%) had been diagnosed 1–8 years previously, 26 (32.5%) 9–17 years previously, and 3 (3.75%) 18 or more years previously.

**Table 1:** Demographic and clinical characteristics of PD patients

**Age (years), mean ± SD**	70.6 ± 8.9	
**Gender, n (%)**		
Female	34 (42.5)	
Male	46 (57.5)	
**Education Level, n (%)**		
Elementary School	3 (3.75)	
Middle School	16 (20)	
High School Diploma	36 (45)	
Bachelor’s Degree	5 (6.25)	
Master’s Degree	20 (25)	
**Physiotherapy, n (%)**		
Yes	39 (48.75)	
No	41 (51.25)	
**Employment Status, n (%)**		
Employed	16 (20)	
Not in employment	64 (80)	
**Time Since Diagnosis (years), n(%)**		
1–8 years	51 (63.75)	
9–17 years	26 (32.5)	
≥ 18 years	3 (3.75)	
**BMI, n (%)**		
Normal Weight	39 (48.75)	
Overweight	41 (51.25)	
**EQ-Index, mean ± SD**	0.641 ± 0.307	
**EQ-VAS, mean ± SD**	59.35 ± 17.32	
**MDS-UPDRS, mean ± SD**		
Part I (*n* = 39)	12.4 ± 6.8	
Part II (*n* = 39)	14.1 ± 8.8	
Part III (*n* = 40)	41.9 ± 19.2	
Part IV (*n* = 40)	4.5 ± 4.6	
**MDS-UPDRS Part III severity, n (%)**		
Mild (≤ 32)	10 (25.0)	
Moderate (33–58)	23 (57.5)	
Severe (≥ 59)	7 (17.5)	
**Mini-Mental Parkinson (** ***n*** ** = 50)**	27.6 ± 3.8	

BMI = body mass index; EQ-Index = EQ-5D-5 L index value; EQ-VAS = EQ-5D-5 L visual analogue scale; MDS-UPDRS = Movement Disorder Society–Sponsored Revision of the Unified Parkinson’s Disease Rating Scale; SD = standard deviation

Among participants with available routine-care assessments, mean MDS-UPDRS scores were 12.4 ± 6.8 for Part I and 14.1 ± 8.8 for Part II (*n* = 39), and 41.9 ± 19.2 for Part III and 4.5 ± 4.6 for Part IV (*n* = 40). Based on the published MDS-UPDRS Part III thresholds proposed by Martínez-Martín and colleagues [37], 10 participants (25.0%) were classified as having mild, 23 (57.5%) moderate, and 7 (17.5%) severe motor impairment. The mean MMP score was 27.6 ± 3.8 among the 50 participants with available data, of whom 16 scored below the conventional cut-off of 27.

In the whole cohort, the mean EQ-Index was 0.641 ± 0.307 and the mean EQ-VAS was 59.35 ± 17.32 (95% CI 55.55–63.15).

### Distribution and interpretability

The EQ-Index showed a markedly negatively skewed distribution (skewness − 1.59; kurtosis 1.85; Shapiro–Wilk *p* < 0.001), while the EQ-VAS also departed from normality (skewness − 0.43; kurtosis − 0.56; Shapiro–Wilk *p* = 0.004). Fifty-nine distinct EQ-5D-5 L health states were observed. One participant reported full health (11111), corresponding to a ceiling proportion of 1.2%, whereas no participant reported the worst possible health state (55555; floor proportion 0%). Six participants (7.5%) had negative EQ-Index values.

### Descriptive inter-dimensional associations

Cronbach’s alpha across the five EQ-5D-5 L dimensions was 0.853 and was 0.848 when calculated using standardised dimensions. Corrected dimension–total correlations ranged from 0.428 for anxiety/depression to 0.815 for usual activities. Deletion of the anxiety/depression dimension increased alpha to 0.875, whereas deletion of any other dimension reduced the coefficient (Table 2). These statistics were interpreted descriptively as measures of association among the dimensions and not as evidence of internal-consistency reliability or a single latent HRQoL construct.

**Table 2:** Descriptive inter-dimensional associations of the EQ-5D-5 L descriptive system

EQ-5D-5 L dimension	Mean ± SD	Corrected dimension–total correlation	Cronbach’s α if dimension deleted
Mobility	2.44 ± 0.939	0.807	0.785
Self-Care	2.23 ± 1.055	0.730	0.804
Usual Activities	2.4 ± 1.13	0.815	0.778
Pain/Discomfort	2.46 ± 0.885	0.565	0.846
Anxiety/Depression	2.29 ± 0.86	0.428	0.875

EQ-5D-5 L = EuroQol five-dimension five-level questionnaire; SD = standard deviation. *n* = 80. Each dimension is scored from 1 (no problems) to 5 (extreme problems or inability, as applicable); higher scores indicate more severe reported problems. Cronbach’s α across the five dimensions was 0.853 and was 0.848 when calculated using standardised dimensions. Corrected dimension–total correlations were calculated between each dimension and the total score formed from the remaining dimensions. Alpha and the related coefficients are reported descriptively as indices of inter-dimensional association and should not be interpreted as evidence of unidimensionality or internal-consistency reliability

### Short-term reproducibility

No statistically detectable baseline differences were observed between retested and non-retested participants in EQ-Index (*p* = 0.939) or EQ-VAS scores (*p* = 0.705).

At dimension level, quadratic-weighted kappa ranged from 0.632 for pain/discomfort — the only coefficient below the 0.70 threshold — to 0.916 for mobility, with exact agreement between 70.0% and 85.0%. Agreement was high for the summary scores: the single-measures ICC was 0.901 (95% CI 0.840–0.940) for the EQ-Index and 0.938 (95% CI 0.899–0.963) for the EQ-VAS. No systematic difference was detected between administrations for either summary score (EQ-Index: paired t *p* = 0.880, Wilcoxon *p* = 0.836; EQ-VAS: *p* = 0.279 and *p* = 0.320). The SEM was 0.096 index points for the EQ-Index and 4.22 points for the EQ-VAS, corresponding to a smallest detectable change of 0.266 and 11.68 respectively (Table 3).

**Table 3:** Test-retest analysis

EQ-5D-5 L	Pre-test (mean ± SD)	Post-test (mean ± SD)	Agreement coefficient (95% CI)	Exact agreement
Item 1 (Mobility)	2.45 ± 0.95	2.43 ± 0.96	κ = 0.916 (0.850–0.963)	85.0%
Item 2 (Self-Care)	2.13 ± 1.05	2.10 ± 1.00	κ = 0.855 (0.770–0.916)	70.0%
Item 3 (Usual Activities)	2.35 ± 1.09	2.37 ± 0.99	κ = 0.835 (0.723–0.913)	70.0%
Item 4 (Pain/Discomfort)	2.55 ± 0.85	2.47 ± 0.83	κ = 0.632 (0.363–0.825)	71.7%
Item 5 (Anxiety/Depression)	2.35 ± 0.82	2.37 ± 0.99	κ = 0.785 (0.650–0.875)	70.0%
EQ Index-Score	0.639 ± 0.306	0.637 ± 0.297	ICC = 0.901 (0.840–0.940)	—
EQ VAS-Score	59.80 ± 16.99	60.63 ± 16.73	ICC = 0.938 (0.899–0.963)	—

CI = confidence interval; EQ-Index = EQ-5D-5 L index value; EQ-VAS = EQ-5D-5 L visual analogue scale; ICC = intraclass correlation coefficient; SD = standard deviation; SDC = smallest detectable change; SEM = standard error of measurement; κ = quadratic-weighted Cohen’s kappa. *n* = 60, retested after a 48-hour interval. Quadratic-weighted kappa is reported for the five ordinal dimensions, and single-measures, two-way random-effects, absolute-agreement ICCs are reported for the continuous summary scores. Coefficients ≥ 0.70 were regarded as satisfactory. SEM and SDC were 0.096 and 0.266 for the EQ-Index and 4.22 and 11.68 for the EQ-VAS, respectively

### Construct validity

All 20 prespecified directional hypotheses were confirmed: the EQ-Index and EQ-VAS were positively associated with both SF-12 component scores and negatively associated with all eight PDQ-39 domains (all *p* < 0.01). The strongest associations were observed with PDQ-39 Mobility (ρ = −0.786 for the EQ-Index and − 0.781 for the EQ-VAS), PDQ-39 Activities of Daily Living (ρ = −0.671 and − 0.685), and the SF-12 Physical Component Summary (ρ = 0.730 and 0.639), supporting convergence with physical health and daily functioning (Table 4).

**Table 4:** A priori hypothesis testing for construct validity of the EQ-5D-5 L

Comparator	Expected association	EQ-Index, Spearman’s ρ	EQ-VAS, Spearman’s ρ	Magnitude confirmed, Index / VAS
SF-12 PCS-12	Positive, strong	0.730**	0.639**	Yes / Yes
SF-12 MCS-12	Positive, moderate	0.541**	0.524**	No / No
PDQ-39 Mobility, items 1–10	Negative, strong	−0.786**	−0.781**	Yes / Yes
PDQ-39 Activities of Daily Living, items 11–16	Negative, strong	−0.671**	−0.685**	Yes / Yes
PDQ-39 Emotional Well-being, items 17–22	Negative, moderate	−0.629**	−0.567**	No / No
PDQ-39 Stigma, items 23–26	Negative, weak	−0.373**	−0.334**	No / No
PDQ-39 Social Support, items 27–29	Negative, weak	−0.374**	−0.376**	No / No
PDQ-39 Cognition, items 30–33	Negative; magnitude not prespecified	−0.468**	−0.491**	—
PDQ-39 Communication, items 34–36	Negative, weak	−0.445**	−0.403**	No / No
PDQ-39 Bodily Discomfort, items 37–39	Negative; magnitude not prespecified	−0.490**	−0.398**	—

EQ-Index = EQ-5D-5 L index value; EQ-VAS = EQ-5D-5 L visual analogue scale; MCS-12 = Mental Component Summary; PCS-12 = Physical Component Summary; PDQ-39 = Parkinson’s Disease Questionnaire-39; SF-12 = 12-item Short Form Health Survey. *n* = 80. Higher EQ-5D-5 L and SF-12 scores and lower PDQ-39 scores indicate better health

**p < 0.01, two-tailed

Among the 16 associations for which both direction and magnitude had been prespecified, 6 (37.5%) met the expected magnitude criterion. Both EQ-5D-5 L summary scores showed the expected strong associations with the SF-12 Physical Component Summary and the PDQ-39 Mobility and Activities of Daily Living domains. Associations with the SF-12 Mental Component Summary and PDQ-39 Emotional Well-being were strong rather than moderate, whereas those with PDQ-39 Stigma, Social Support, and Communication were moderate rather than weak. Associations with PDQ-39 Cognition and Bodily Discomfort were in the expected negative direction but were interpreted descriptively because no magnitude hypotheses had been prespecified for these domains. Overall, the findings supported convergence with physical-health constructs, whereas the hypothesised discrimination from conceptually more distant domains was not fully supported. Pearson sensitivity analyses produced the same directional pattern, with 7 of the 16 prespecified magnitude criteria met (Supplementary Table S1).

### Associations with routinely recorded clinical measures

In post hoc available-case analyses, higher MDS-UPDRS scores were consistently associated with poorer health status on both EQ-5D-5 L summary measures (Table 5). Moderate-to-strong inverse correlations were observed for MDS-UPDRS Parts I–III, with the strongest association found between Part II and the EQ-VAS (ρ = −0.792). Associations with Part IV were weaker, although they remained statistically significant for both the EQ-Index and EQ-VAS. MMP scores were not associated with either the EQ-Index (ρ = 0.022) or the EQ-VAS (ρ = 0.100). Given the post hoc nature of these analyses and the incomplete availability of routine-care clinical data, these findings were interpreted as exploratory.

**Table 5:** Post hoc associations between EQ-5D-5 L summary scores and routinely recorded clinical measures

Clinical measure	*n*	EQ-Index, Spearman’s ρ	EQ-VAS, Spearman’s ρ
MDS-UPDRS Part I	39	−0.627**	−0.522**
MDS-UPDRS Part II	39	−0.644**	−0.792**
MDS-UPDRS Part III	40	−0.593**	−0.694**
MDS-UPDRS Part IV	40	−0.391*	−0.440**
Mini-Mental Parkinson	50	0.022	0.100

EQ-Index = EQ-5D-5 L index value; EQ-VAS = EQ-5D-5 L visual analogue scale; MDS-UPDRS = Movement Disorder Society–Sponsored Revision of the Unified Parkinson’s Disease Rating Scale

Reported p values are unadjusted and two-tailed. *p < 0.05; **p < 0.01

### Clinical discrimination across motor-severity categories

Among participants with available MDS-UPDRS Part III data, 10 were classified as having mild, 23 moderate, and 7 severe motor impairment according to the externally established thresholds. Both EQ-5D-5 L summary scores decreased monotonically with increasing motor severity (Table 6). Mean EQ-Index values declined from 0.850 in the mild group to 0.661 in the moderate group and 0.330 in the severe group; the corresponding EQ-VAS values were 74.0, 59.3, and 42.9. The ordered trend was statistically significant for both outcomes (Jonckheere–Terpstra z = − 4.10 and − 4.15, respectively; both *p* < 0.001) and was corroborated by the Kruskal–Wallis and one-way ANOVA results.

**Table 6:** Post hoc comparison of EQ-5D-5 L summary scores across externally defined MDS-UPDRS Part III motor-severity categories

MDS-UPDRS Part III category	*n*	EQ-Index, mean ± SD; median [IQR]	EQ-VAS, mean ± SD; median [IQR]
***Descriptive statistics***			
Mild (≤ 32)	10	0.850 ± 0.085; 0.862 [0.776–0.909]	74.0 ± 8.1; 72.5 [70.0–78.8]
Moderate (33–58)	23	0.661 ± 0.290; 0.717 [0.684–0.788]	59.3 ± 15.1; 60.0 [50.0–70.0]
Severe (≥ 59)	7	0.330 ± 0.338; 0.279 [0.112–0.614]	42.9 ± 12.2; 45.0 [35.0–50.0]
***Inferential analyses***			
Analysis		EQ-Index	EQ-VAS
Jonckheere–Terpstra test		z = − 4.10; *p* < 0.001	z = − 4.15; *p* < 0.001
Kruskal–Wallis test		H = 15.67; *p* < 0.001	H = 16.49; *p* < 0.001
One-way ANOVA		F = 7.94; *p* = 0.001	F = 11.46; *p* < 0.001
η²		0.30	0.38
Mild–severe mean difference (95% CI)		0.520 (0.264 to 0.775)	31.1 (20.8 to 41.5)
Mild–severe Hedges’ g (95% CI)		2.21 (0.96 to 3.45)	2.97 (1.54 to 4.41)

CI = confidence interval; EQ-Index = EQ-5D-5 L index value; EQ-VAS = EQ-5D-5 L visual analogue scale; IQR = interquartile range; MDS-UPDRS = Movement Disorder Society–Sponsored Revision of the Unified Parkinson’s Disease Rating Scale; SD = standard deviation. Descriptive values are reported as mean ± SD and median [IQR]. Higher EQ-5D-5 L scores indicate better health. Motor-severity thresholds were derived externally from Martínez-Martín et al. [37] and were not generated from the present data. The Jonckheere–Terpstra test was the principal analysis because the severity categories were ordered and a decreasing trend in EQ-5D-5 L scores was expected. Kruskal–Wallis and one-way ANOVA results are reported as supplementary omnibus analyses. No expected effect magnitude was prespecified. The analysis was post hoc, and the severe group included seven participants

The observed between-group effect estimates were large: η² was 0.30 for the EQ-Index and 0.38 for the EQ-VAS. Compared with the severe group, the mild group scored 0.520 index points higher (95% CI 0.264 to 0.775; Hedges’ g = 2.21) and 31.1 EQ-VAS points higher (95% CI 20.8 to 41.5; Hedges’ g = 2.97). These estimates should nevertheless be interpreted cautiously because the analysis was post hoc and the severe group included only seven participants.

### Exploratory subgroup analyses

Exploratory subgroup comparisons are summarised in Table 7. Participants in employment had higher EQ-Index and EQ-VAS scores than those not in employment (Mann–Whitney *p* = 0.010 and *p* = 0.049, respectively). Time since diagnosis, analysed as a continuous variable, was inversely associated with both the EQ-Index (Spearman’s ρ = −0.377, *p* = 0.001) and the EQ-VAS (ρ = −0.451, *p* < 0.001). No differences were observed according to gender.

**Table 7:** Exploratory subgroup comparisons of EQ-5D-5 L summary scores

Characteristic	*n*	EQ-Index, mean ± SD	Primary *p* value	Sensitivity *p* value	EQ-VAS, mean ± SD	Primary *p* value	Sensitivity *p* value
**Gender**			0.474	0.229ᵃ		0.517	0.421
Female	34	0.591 ± 0.348			61.18 ± 16.00		
Male	46	0.678 ± 0.271			58.00 ± 18.29		
**Employment status**			0.010	< 0.001ᵃ		0.049	0.015ᵃ
Employed	16	0.806 ± 0.090			66.88 ± 11.67		
Not in employment	64	0.599 ± 0.328			57.47 ± 18.05		
**Educational level**			0.508	0.019		0.153	0.091
Elementary school	3	0.391 ± 0.537			46.66 ± 25.17		
Middle school	16	0.445 ± 0.443			50.93 ± 20.26		
High-school diploma	36	0.722 ± 0.176			63.41 ± 11.87		
Bachelor’s degree	5	0.693 ± 0.321			65.00 ± 24.49		
Master’s degree	20	0.674 ± 0.252			59.25 ± 18.58		
**Physiotherapy**			0.016	0.054		0.377	0.594
Yes	39	0.573 ± 0.339			58.28 ± 16.18		
No	41	0.705 ± 0.261			60.37 ± 18.49		
**BMI category**			0.050	0.022		0.680	0.508
Normal weight	39	0.560 ± 0.371			58.03 ± 18.47		
Overweight	41	0.717 ± 0.207			60.61 ± 16.29		

BMI = body mass index; EQ-Index = EQ-5D-5 L index value; EQ-VAS = EQ-5D-5 L visual analogue scale; SD = standard deviation. *n* = 80. Higher EQ-5D-5 L scores indicate better health. Primary analyses were conducted using the Mann–Whitney U test for two-level grouping variables and the Kruskal–Wallis test for educational level. Independent-samples Student’s t-tests and one-way analysis of variance were conducted as parametric sensitivity analyses. ᵃWelch’s correction was applied because the assumption of equal variances was not met. Time since diagnosis was analysed as a continuous variable using Spearman’s rank correlation coefficient and is reported in the text. No adjustment for multiple comparisons was applied. Comparisons for which the primary and sensitivity analyses led to different conclusions were considered inconclusive. These analyses were exploratory and were not used as confirmatory evidence of known-groups validity

For the EQ-Index, educational level, physiotherapy engagement, and BMI produced discordant conclusions between the primary non-parametric analyses and the corresponding parametric sensitivity analyses and were therefore considered inconclusive. The corresponding EQ-VAS comparisons were non-significant under both analytical approaches. Because these analyses were exploratory, were not based on prespecified directional hypotheses, and were not adjusted for multiple comparisons, none was used as confirmatory evidence of known-groups validity.

## Discussion

This study evaluated selected measurement properties of the Italian EQ-5D-5 L in people with PD. The EQ-Index and EQ-VAS showed high short-term reproducibility. All 20 prespecified directional hypotheses were confirmed, whereas only 6 of the 16 associations for which an expected magnitude had also been specified met that criterion. This pattern provided stronger support for convergence with physical functioning and daily activities than for discrimination from more conceptually distant HRQoL domains. Post hoc analyses also showed an ordered decline in EQ-5D-5 L scores across motor-severity categories, although these findings remain exploratory. The results should therefore be interpreted as evidence concerning selected measurement properties rather than as a comprehensive validation of the instrument.

Cronbach’s alpha across the five dimensions was 0.853, close to the value of 0.828 reported in a large Mexican PD cohort [15]. This similarity should be interpreted descriptively rather than as evidence that the EQ-5D-5 L forms a cohesive unidimensional scale. Meaningful interpretation of alpha as internal-consistency reliability assumes a reflective measurement model, whereas previous research suggests that the relationships among the EQ-5D-5 L dimensions may combine formative and reflective components [38]. The comparatively low corrected dimension–total correlation for anxiety/depression and the increase in alpha following its deletion further illustrate the limitations of a conventional reflective interpretation. Accordingly, these coefficients were used only to describe inter-dimensional associations and not to infer internal-consistency reliability or structural validity.

Short-term reproducibility was high for the EQ-Index and EQ-VAS, whereas agreement was less uniform at the dimension level, with pain/discomfort falling below the prespecified threshold. This pattern is broadly consistent with findings reported for the EQ-5D-5 L in other neurological populations, including stroke and multiple sclerosis [11, 21], although the methodological constraints of the retest design warrant cautious interpretation and are considered below.

The construct-validity findings were mixed. The strongest associations involved the SF-12 Physical Component Summary and the PDQ-39 Mobility and Activities of Daily Living domains, a pattern that is conceptually coherent given their overlap with the mobility, self-care, and usual-activities dimensions of the EQ-5D-5 L. By contrast, associations with mental and emotional constructs were stronger than anticipated, while those with Stigma, Social Support, and Communication were moderate rather than weak. The negative associations with Cognition and Bodily Discomfort were also directionally consistent, but their magnitudes were interpreted descriptively because no magnitude criteria had been prespecified for these domains. Taken together, the findings provide clearer support for convergent validity with physical functioning and independence than for discriminant validity across more conceptually distant HRQoL domains. The limited separation between constructs may reflect the broad and interrelated effects of PD across physical, emotional, and social functioning, as well as shared method variance among self-reported measures [29, 39]. Nevertheless, the convergence with physical-functioning domains is consistent with previous EQ-5D studies in PD [15, 16].

Content validity was not evaluated in the present cohort. However, PD-specific qualitative evidence has identified several domains important to people with PD—including dexterity, fatigue, sleep, dyskinesia, and bowel or bladder problems—that are incompletely represented across generic preference-based measures, including the EQ-5D-5 L [40]. This evidence reinforces the complementary role of the EQ-5D-5 L: its generic preference-based profile and utility score should be used alongside, rather than in place of, disease-specific clinical and patient-reported measures.

The post hoc clinical analyses provided complementary, although exploratory, evidence that EQ-5D-5 L scores reflect clinically relevant disease burden. All four MDS-UPDRS parts were inversely associated with the EQ-Index and EQ-VAS, with the strongest association observed between Part II and the EQ-VAS. This finding is conceptually coherent because motor experiences of daily living overlap with the mobility, self-care, and usual-activities dimensions of the EQ-5D-5 L, and is consistent with evidence from a large cohort of people with PD in which greater MDS-UPDRS Part II impairment remained independently associated with a lower EQ-Index. The monotonic decline in both summary scores across mild, moderate, and severe MDS-UPDRS Part III categories provides clinically coherent post hoc evidence that poorer EQ-5D-5 L health status accompanied greater motor impairment in the assessed subsample. MMP scores were not associated with either summary measure; however, this finding should not be interpreted as evidence that cognition is unrelated to HRQoL, as the association between cognitive impairment and EQ-5D-5 L scores reported in previous research was statistically significant but comparatively small [41]. Accordingly, these analyses should be interpreted as supportive, hypothesis-generating evidence that EQ-5D-5 L scores reflect clinically relevant disease burden, rather than as confirmatory evidence of construct validity.

The exploratory associations with employment status and disease duration warrant cautious interpretation. Employment may act as a proxy for preserved functional independence, lower disease burden, younger age, or socioeconomic resources rather than representing an independent determinant of HRQoL. Likewise, disease duration is an imperfect surrogate for current clinical severity, as progression and functional impairment vary considerably among people with PD [42–44]. Because these analyses were not hypothesis-driven, were not adjusted for potential confounders or multiple comparisons, and some findings were sensitive to the analytical approach used, they should not be regarded as confirmatory evidence of known-groups validity. Rather, they identify potentially relevant relationships that should be examined prospectively using prespecified hypotheses, standardised severity measures, and multivariable models.

Beyond these psychometric findings, a practical strength of the EQ-5D-5 L is that, unlike the PDQ-39, it positions PD on a metric common to other conditions and generates utility values for cost-utility analysis and the estimation of quality-adjusted life-years, making it particularly valuable for group-level research and health-policy decision-making [45–47]. The five-level descriptive system was developed to reduce ceiling effects and improve discriminatory capacity compared with the EQ-5D-3 L [12, 48]. In the present sample, only one participant reported full health and 59 distinct health states were observed, indicating substantial descriptive variation across the health profiles represented.

### Limitations

Several limitations should be considered when interpreting these findings. First, the study evaluated selected measurement properties rather than providing a comprehensive validation of the Italian EQ-5D-5 L. Structural validity and cross-cultural validity or measurement invariance were not examined, while criterion validity was not assessed because no accepted gold standard exists for HRQoL. Content validity was not evaluated in the present cohort, although the available PD-specific evidence and its implications for the complementary use of the EQ-5D-5 L have been discussed above. Moreover, the cross-sectional design precluded the assessment of responsiveness, longitudinal validity, and the minimal important change. The present findings therefore do not support the use of the instrument for monitoring change in individual patients.

Second, the single-centre clinical setting and the absence of a prospective screening log limit assessment of the representativeness and generalisability of the cohort. Although enrolment was consecutive and all enrolled participants completed the primary PROMs, the number of patients assessed for eligibility and the reasons for non-enrolment could not be reconstructed. The post hoc clinical analyses also relied on routine-care MDS-UPDRS and MMP data that were available for only part of the cohort; selection bias related to the availability of these assessments therefore cannot be excluded. In addition, the motor-severity groups were small, particularly the severe group, which included seven participants, and Hoehn and Yahr staging was unavailable.

Third, cognitive screening was not incorporated prospectively into the eligibility assessment. Routine MMP data were available for only 50 participants, 16 of whom scored below the conventional cut-off of 27. Because a numerical threshold could not be applied consistently across the entire cohort, the possible influence of unrecognised cognitive impairment on questionnaire comprehension and self-report cannot be excluded, despite the clinical assessment of participants’ ability to complete the questionnaires independently.

Fourth, the retest subsample comprised the first 60 participants enrolled rather than a randomly selected subgroup. Although the 48-hour interval reduced the likelihood of genuine clinical change, it may have increased recall of previous responses, and no external criterion of clinical stability was administered. The agreement coefficients should therefore be interpreted as estimates of short-term reproducibility and may overestimate reproducibility over longer intervals. Finally, the demographic and clinical subgroup comparisons were exploratory, were not based on prespecified directional hypotheses, and were not adjusted for multiple comparisons. Findings that differed between the primary non-parametric analyses and the corresponding parametric sensitivity analyses were considered inconclusive and were not used to support the construct-validity conclusions.

Future studies should use larger, multicentre and longitudinal cohorts, maintain prospective screening logs, collect standardised motor-severity and cognitive measures in all participants, incorporate an external stability criterion into reproducibility analyses, and evaluate responsiveness and the minimal important change.

## Conclusions

The Italian EQ-5D-5 L showed high short-term reproducibility for the EQ-Index and EQ-VAS, although agreement was less uniform at the dimension level, and provided evidence supporting selected aspects of construct validity in people with PD, particularly convergence with physical functioning and daily activities. Discrimination from conceptually more distant HRQoL domains was less clearly supported. The associations with routinely recorded clinical measures and the ordered decline across motor-severity categories were clinically coherent but derived from post hoc analyses and require prospective confirmation. These findings do not constitute a comprehensive validation, as responsiveness, content validity, structural validity, cross-cultural validity or measurement invariance, and criterion validity were not evaluated. The EQ-5D-5 L may therefore complement disease-specific measures in group-level research and health-economic evaluation; however, larger multicentre longitudinal studies are needed before it can be recommended for monitoring change in individual patients.

## Supplementary Information

Below is the link to the electronic supplementary material.

Supplementary Material 1

## Data Availability

The datasets generated and/or analysed during the current study are available from the corresponding author upon reasonable request, subject to a substantiated scientific rationale.

## Abbreviations

15D: 15-dimensional health-related quality-of-life instrument
ANOVA: Analysis of variance
BMI: Body mass index
CI: Confidence interval
COSMIN: COnsensus-based Standards for the selection of health Measurement Instruments
EQ-5D: EuroQol five-dimension questionnaire
EQ-5D-3L: EuroQol five-dimension three-level questionnaire
EQ-5D-5L: EuroQol five-dimension five-level questionnaire
EQ-Index: EQ-5D-5 L index value
EQ-VAS: EuroQol visual analogue scale
HRQoL: Health-related quality of life
ICC: Intraclass correlation coefficient
IQR: Interquartile range
MCS-12: Mental Component Summary score
MDS-UPDRS: Movement Disorder Society–Sponsored Revision of the Unified Parkinson’s Disease Rating Scale
MMP: MINI-Mental Parkinson
PbQoL: Preference-based quality of life
PCS-12: Physical Component Summary score
PD: Parkinson’s disease
PDQ-8: Parkinson’s Disease Questionnaire-8
PDQ-39: Parkinson’s Disease Questionnaire-39
PROM: Patient-reported outcome measure
SD: Standard deviation
SDC: Smallest detectable change
SEM: Standard error of measurement
SF-12: 12-item Short Form Health Survey
SF-36: 36-item Short Form Health Survey
