# Supplementary material for: Reliability and construct validity of the Italian EQ-5D-5 L in Parkinson’s disease: a cross-sectional study
Source: J Patient Rep Outcomes. 2026 Aug 10;10:202. doi: 10.1186/s41687-026-01174-2 (PMC13740398; doi:10.1186/s41687-026-01174-2)
Supplement: Supplementary file 1 [file 41687_2026_1174_MOESM1_ESM.docx]

**Supplementary Table S1.** Pearson sensitivity analyses for construct validity

| Comparator | EQ-Index, Pearson’s r | EQ-VAS, Pearson’s r |
| --- | --- | --- |
| SF-12 PCS-12 | 0.607** | 0.602** |
| SF-12 MCS-12 | 0.463** | 0.518** |
| PDQ-39 Mobility | −0.700** | −0.726** |
| PDQ-39 Activities of Daily Living | −0.685** | −0.703** |
| PDQ-39 Emotional Well-being | −0.587** | −0.539** |
| PDQ-39 Stigma | −0.402** | −0.394** |
| PDQ-39 Social Support | −0.470** | −0.411** |
| PDQ-39 Cognition | −0.496** | −0.523** |
| PDQ-39 Communication | −0.443** | −0.408** |
| PDQ-39 Bodily Discomfort | −0.435** | −0.361** |

EQ-Index = EQ-5D-5L index value; EQ-VAS = EQ-5D-5L visual analogue scale; MCS-12 = Mental Component Summary; PCS-12 = Physical Component Summary; PDQ-39 = Parkinson’s Disease Questionnaire-39; SF-12 = 12-item Short Form Health Survey. n = 80. Higher EQ-5D-5L and SF-12 scores and lower PDQ-39 scores indicate better health. This table reports the parametric sensitivity analyses corresponding to the primary Spearman analyses presented in Table 4.

**p < 0.01, two-tailed.
